# Clinical impact of delays in the management of lung cancer patients in the last decade: systematic review

**DOI:** 10.1007/s12094-022-02796-w

**Published:** 2022-03-07

**Authors:** María Guirado, Elena Fernández Martín, Alberto Fernández Villar, Arturo Navarro Martín, Alfredo Sánchez-Hernández

**Affiliations:** 1 Medical Oncology Department, Hospital General Universitario de Elche, 03203 Elche, Spain; 2 Thoracic Surgery Department, Hospital Clinico San Carlos, Madrid, Spain; 3 Pneumology Department, Hospital Álvaro Cunqueiro de Vigo, Instituto de Investigación Sanitaria de Vigo, Vigo, Spain; 4 Radiation Oncology Department, Hospital Durani Reynals, Hospitalet, L’Hospitalet de Llobregat, Barcelona, Spain; 5 Medical Oncology Department, Consorcio Hospitalario Provincial de Castellón, 12002 Castellon, Spain

**Keywords:** Lung cancer, Timeliness of care, Delays, Prognosis, Survival, Systematic literature review

## Abstract

**Introduction:**

Due to the importance of lung cancer early treatment because of its severity and extent worldwide a systematic literature review was conducted about the impact of delays in waiting times on the disease prognosis.

**Materials and Methods:**

We conducted a systematic search of observational studies (2010-2020) including adult patients diagnosed with lung cancer and reporting healthcare timelines and their clinical consequences.

**Results:**

We included 38 articles containing data on waiting times and prognosis; only 31 articles linked this forecast to a specific waiting time. We identified 41 healthcare time intervals and found medians of 6-121 days from diagnosis to treatment and 4-19.5 days from primary care to specialist visit: 37.5% of the intervals indicated better prognosis with longer waiting times.

**Conclusions:**

All articles emphasized that waiting times must be reduced to achieve good management and prognosis of lung cancer. Further prospective studies are needed on the relationship between waiting times and prognosis of lung cancer.

## Introduction

Lung cancer, the leading cause of cancer death worldwide, is a health problem of the first order due to the morbidity and mortality caused, and the economic impact it has on health systems [1, 2].

Diagnostic suspicion of early-stage lung cancer may be difficult because the clinical presentation is silent in the early stages and the differential diagnosis may be confusing in advanced stages. Progressive improvements in local and remote diagnostic techniques (EBUS and PET-TAC) and therapeutic advances (targeted therapies, immunotherapies, etc.) in the last decade have improved the prognosis in patients with lung cancer in advanced countries, including Spain. Five-year survival is between 12 and 18% [3] and is directly related to the stage at presentation and the histology: the 5-year survival of patients with localized stages of the disease ranges between 27% and 63%, in regional stages between 16 and 35% and in disseminated stages between 3 and 7%, with the lowest survival rate corresponding to small cell lung cancer (SCLC) [4]. In 2020, 1,796,144 deaths worldwide and 22,930 deaths in Spain were due to lung cancer [3, 5].

The clinical management of lung cancer patients requires complex coordination by specialized medical and surgical services, health service administrators, care managers and social service providers. The traditional approach of referring patients to different specialist consultations sequentially often results in care that is perceived as slow, fragmented, and poorly coordinated. To reduce these delays, agreed standards have been established for maximum acceptable waiting times for lung cancer-specific referral, diagnosis, and treatment times based on expert clinical opinion [1, 6, 7].

In the United Kingdom, the National Optimal Lung Cancer Pathway guidelines propose care algorithms to be used in conjunction with the British Thoracic Society (BTS) and the National Institute for Health and Care Excellence (NICE) guidelines, with the aim of achieving maximum times of 14 days for diagnosis and 28 days for treatment. However, these standards are not always met and delays in lung cancer care persist [1].

It is essential to obtain optimal clinical results in patients with suspected lung cancer to speed up the diagnostic process and early treatment as much as possible. Delays in any part of the process, from the initial evaluation and referral to the definitive diagnosis, treatment and follow-up may have negative consequences [1, 6, 8, 9].

Considering the importance of an early approach in the diagnosis and treatment of lung cancer, we carried out a systematic literature review (SLR) to determine the evidence of the impact of delays in the times of diagnosis and initial treatment on the disease prognosis.

## Materials and methods

The SLR was carried out according to the Preferred Reporting Items for Systematic Reviews and Meta-analyses Statement (PRISMA).

We selected observational studies of patients aged ≥ 18 years diagnosed with or with a clinical suspicion of small cell or non-small cell lung cancer conducted in Europe, the United States, Canada, Japan, Australia, New Zealand, and China. The study had to evaluate ≥ 1 variable related to healthcare deadlines and their effect on clinical outcomes. Randomized clinical trials were not included.

Two search strategies were designed, one for MEDLINE (through PubMed) and one for EMBASE, in which terms related to lung cancer, healthcare deadlines (waiting times, delays, early diagnosis, etc.) and clinical outcomes (prognosis, survival, mortality, etc.) were used. The time horizon of the search was January 1, 2010-November 24, 2020, to include advances in the last decade in the diagnosis and treatment of lung cancer. The language of the publications was limited to English and Spanish.

The titles and abstracts resulting from the search, after duplicate articles were removed, were evaluated by three reviewers (AGC, IAF, and MCA), and those that did not meet the inclusion and exclusion criteria were ruled out, noting the specific reasons. If there was disagreement between reviewers regarding the inclusion of an article, the criterion of a fourth reviewer (FIO) was used. A complete reading of the articles was made by three reviewers (AGC, IAF, and MCA) independently, and the reasons for non-selection were recorded.

Data from the selected articles were tabulated by three reviewers (AGC, IAF, and MCA) on a form developed specifically for extraction and validated by a fourth reviewer. From each article selected, we extracted the study characteristics (type of study, design, country of study, sample size, study duration, follow-up time), patient characteristics (mean age, sex ratio, disease stage), healthcare deadlines (time intervals evaluated between symptoms, diagnosis, and treatment [including mean, standard deviation, median or interquartile range]) and clinical outcomes (survival, mortality). All waiting time intervals were analyzed in calendar days (if an article reported delays in weeks, these values were multiplied by 7; if it reported delays in months, they were multiplied by 30.41).

All results focused on the healthcare timelines of lung cancer patients and their clinical consequences were evaluated.

The times evaluated were expressed as: (a) time from the appearance of symptoms or clinical or radiological suspicion (first abnormal imaging test) to the therapeutic intervention, (b) partial times, considering: (b1) time from the appearance of symptoms, clinical or radiological suspicion to diagnosis (lung cancer study, staging), (b2) time from diagnosis to therapeutic decision, (b3) time from therapeutic decision to treatment initiation. Clinical outcomes related to the prognosis were progression-free survival, overall survival, time to relapse, and mortality.

Initially, given the variations in the times considered and to interpret the information in the most aggregated and homogeneous way possible, the time intervals were grouped sequentially following the timeline that goes from diagnosis to treatment. The groups of time intervals evaluated were described using absolute frequencies (*n*) and percentages with respect to the total number of articles selected.

To evaluate the relationship between the time intervals stated and their association with the prognosis, we made a qualitative analysis that categorized the possible relationship between the time intervals and the specific prognosis in relation to survival and mortality.

## Results

**Fig. 1:**
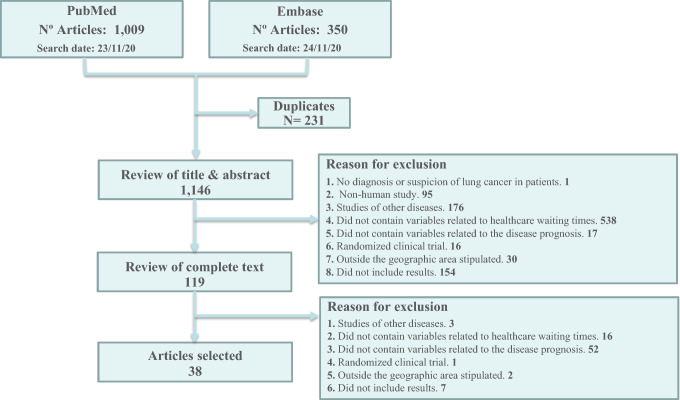
PRISMA flowchart

The search strategy and the decisions made during the selection of the articles included in the SLR are shown in the PRISMA flowchart (Fig. 1). The search identified 1359 articles for review, of which, after eliminating duplicate articles, 1146 were assessed for eligibility based on the title and abstract; of these, 1027 were excluded, mostly because they did not provide variables related to waiting times. The full text of the remaining 119 articles was evaluated, and 81 were excluded, mainly because they did not include variables related to the disease prognosis (*n* = 52) or waiting times (*n* = 16). Finally, 38 met the inclusion criteria.

### Description of the studies

Thirty-four studies were retrospective observational studies, three were prospective observational studies, and one was a systematic literature review.

The studies included 1,225,328 patients, with a sample size ranging from 128 to 691,464. Twenty-one studies were conducted specifically in patients with non-small cell lung cancer (NSCLC), 13 in patients with any type of lung cancer, 3 in patients with SCLC, and one in patients with epidermoid NSCLC. Twenty-one studies investigated all disease stages, four included only patients with stage I–III, three included stage III and IV patients, three studies only included stage I patients, two studies included stage I and II patients, one study only included stage III patients, and another included only stage II and III, three studies did not specify this information.

There were wide variations and heterogeneity among the studies included. The quality was evaluated using the National Heart, Lung and Blood Institute tool. One of the most common shortcomings was the lack of justification of the number of patients needed to detect a relationship between the waiting time and the prognosis, and the statistical adjustment of the variables influencing the prognosis.

The characteristics of the studies selected are shown in Table 2.

### Description of times

**Fig. 2:**
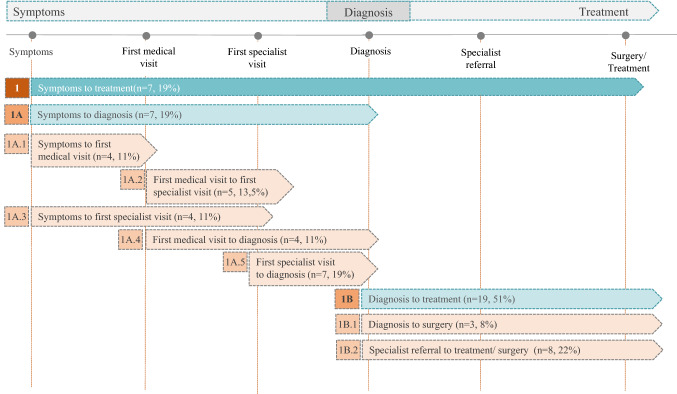
Time intervals according to the specifications of the articles selected

In the 38 selected articles, the results of 41 healthcare time intervals were originally described, which conditioned the type of analysis. The most common waiting times were from symptoms to treatment (7 articles, 19%), symptoms to diagnosis (7 articles, 19%), first specialist visit to diagnosis (7 articles, 19%), specialist referral to surgery or treatment (8 articles, 22%) and from diagnosis to treatment (19 articles, 51%) (Fig. 2). The median of the times studied was two time periods in the same study (IQR 1–4), with a maximum of 10. There were wide variations in how the results of the healthcare deadlines were summarized statistically including means, medians, minimum-maximums, and percentage of patients with delays in the time interval studied.

### Description of healthcare time intervals

In articles that studied the time from symptoms to treatment in patients with lung cancer stages I–IV, the median (range) waiting time was 87.5 days (44–130.5). In patients with SCLC stages I–IV, one study reported the median waiting time was 78 days. In patients with NSCLC stages I–IV, the mean was 138.5 days and in patients without a definite stage the median was 62 days. Kuroda et al. [10] defined delay as a wait of > 6 months after diagnosis and until surgical treatment and found that, in patients with NSCLC stage IA, the mean waiting time was 411 days in patients treated in < 2 years, compared with 1669.9 days in patients whose waiting time was > 2 years.

Studies that directly assessed the time from symptoms to diagnosis reported mean or median waiting times of > 20 days. In patients with lung cancer stages I–IV, the median (range) waiting time was 33 days (23–66), and in patients with lung cancer where the stage was not specified, the median was 56 days. The median time was 69 days in patients with SCLC stages I–IV, and 75 days in patients with NSCLC. Concannon et al. [11] distinguished between patients with NSCLC stages I–II who were homeless (mean waiting time of 248 days) and those who had a home (mean waiting time of 116 days), and patients with NSCLC stages III–IV who were homeless (mean waiting time of 34.7 days) and those who had a home (mean waiting time of 46 days).

Several studies evaluated the means and medians of specific waiting times for different subintervals within the symptoms to diagnosis time, specifically:

- For the symptoms to first specialist visit time, the median (range) waiting time was 33.25 days (8–53) for patients with lung cancer stages I–IV, and the mean of the means was 53.33 days.
- For the symptoms to first medical visit time, the mean of the means of waiting time for patients with lung cancer without stage specification was 44.52 days, and for patients with lung cancer stages I–IV, one study reported a median of 58 days. For patients with SCLC stages I–IV, the median reported by one study was 30 days and a mean of 56.7 days in patients with stages I–IV NSCLC was found by another study.
- For the first medical visit to first specialist visit, the median of the medians of waiting time for patients with lung cancer stages I–IV was 5 days. One study reported a mean of 14.49 days in lung cancer patients without specifying the stage. For patients with SCLC and stages I–IV NSCLC, the median was 19.5 and 17 days, respectively.
- For the first medical visit to diagnosis, the mean waiting time for lung cancer patients in whom the stage was not specified was 29.54 days in the study by Zicovic et al. [12] and a median of 88 days in the study by Redaniel et al. [13]. In patients with SCLC and stages I–IV NSCLC, the median was 34 and 40 days, respectively.
- For the first specialist visit to diagnosis, the median of the medians of waiting time in patients with lung cancer stages I–IV was 19.5 days, and the mean was 16.59 days in patients in whom the stage was not specified. In patients with SCLC stages I–IV, the median was 21 days and in patients with NSCLC stages I–IV the mean was 51.3 days.

For the diagnosis to treatment, the median of the medians of waiting time for patients with lung cancer stages I–IV was 31 days, and for patients in stage I–IIIA the mean was 35 days. Forrest et al. [14] indicated that 39.5% of patients had a delay in this time (defining delay as > 31 days from diagnosis to treatment). They also evaluated the time from referral to the specialist until treatment (defining delay as > 14 days) and found that 69.3% of patients had a delay. Samson et al. [15], defined a wait ≥ 8 weeks as a delay of treatment, and studied patients diagnosed with NSCLC stage I, finding that the median time of patients who waited less than 8 weeks from diagnosis to treatment was 29 days, and that of patients who waited 8 weeks or more was 77 days. In patients with NSCLC stage III, Rice et al. [16] distinguished between patients with private insurance, those with basic coverage and those without insurance. The mean waiting times were 25, 48 and 52 days, respectively, and a waiting time > 30 days was considered a delay. In patients with NSCLC stages I–II, the median of the medians of waiting time was 36.55 days. In patients with NSCLC stages I–III, the median wait between diagnosis and treatment was 28 days. In patients with NSCLC stages I–IIIB and IIIB–IV, the median was 121.6 days and 21 days, respectively. In patients with NSCLC stages I–IV, the median of medians was 33.5 days, in agreement with the study by Concannon et al. [11] in patients with NSCLC stages I–II who were homeless, in whom a median waiting time of 20 days and of 50 days in those with homes was reported. In patients with NSCLC stages III–IV without a home the mean was 49.9 days compared with 58.1 days in those with a home. Anggondowati et al. [17] distinguished according to disease progression, reporting median waiting times of 18 days for patients with metastases, 28 days for patients in the early stages and 27 days for patients in locally advanced stages. In patients with stages I–IV SCLC, the median of the medians of diagnosis to treatment was 7.5 days, while Bhandari et al. [18] found a mean of 18 days in these patients.

Three time periods that could not be grouped into any of the previously defined groups were identified: from the decision on surgery to the time of surgery, from diagnosis to contact with the specialist and from surgery to adjuvant treatment. The results were:

- For the decision on surgery to surgery, the percentage of patients with stages I to II NSCLC whose waiting time was < 1 month was 24.8%, between 1 and 2 months 44.1%, between 2 and 3 months 19% and between 3 and 4 months 11.7%.
- For the diagnosis to contact with the specialist, the median of medians in patients with lung cancer stages I–IV was 9 days, while Kanarek et al. [19] found the mean for NSCLC stages I–II patients was 61.2 days: in this study the surgeon was the specialist physician, after diagnosis by the oncologist.
- For the surgery to systemic treatment or vice versa, the median waiting time in patients with NSCLC stages I–III was 48 days, and in patients with stages I–IV 56 days. Odell et al. [20] defined delay as > 120 days from chemotherapy to surgery and 180 days from surgery to chemotherapy: the percentage of patients with NSCLC stages I–IV with a delay, was 4% and 64%, respectively.

### Relationship between healthcare waiting times and the prognosis

The 38 articles included reported, in addition to waiting times, the results related to the prognosis and 31 related the prognosis to a specific healthcare time evaluated. In general, there were wide variations in the results observed with respect to the prognosis in relation to the type of lung cancer studied, the stage and the time interval evaluated (Tables 1, 3).

**Table 1:** Association between waiting times and survival

Time intervals	Number of articles	Association	References
Symptoms to treatment	2	No association between delay and prognosis	[21, 22]
	3	Longer waiting times improve the results forecast	[10, 23, 24]
Symptoms to first specialist visit	2	No association between delay and prognosis	[23, 25]
Symptoms at first medical visit	1	Shorter waiting times improve the results forecast	[26]
	2	No association between delay and prognosis	[21, 22]
	1	Longer waiting times improve the results forecast	[12]
Symptoms to diagnosis	1	Longer waiting times improve the results forecast	[21]
	2	No association between delay and prognosis	[11, 27]
First medical visit to diagnosis	1	Longer waiting times improve the results forecast	[13]
	1	Shorter waiting times improve the results forecast	[12]
First specialist visit to diagnosis	3	No association between delay and prognosis	[12, 25, 28]
Diagnosis to treatment	3	No association between delay and prognosis	[11, 28, 29]
	9	Longer waiting times improve the results forecast	[14, 18, 21–23, 25, 26, 30, 31]
	9	Shorter waiting times improve the results forecast	[15, 17, 19, 32–37]

For the symptoms to treatment time, two studies reported no association between waiting time and survival or mortality, although Alanen [21] found improved survival when the waiting time was shorter in stage I patients. Three studies reported better patient survival when the waiting time was longer, although they justified these results by indicating that, in patients in earlier stages of the disease, the diagnostic study and assessment of staging may be more complex and require more tests, which could extend the times, compared with patients whose disease is more advanced.

For the symptoms to diagnosis time, two studies found no association with survival or mortality and one study reported improved survival when the waiting time was longer, associating this outcome with patients whose only possible treatment is palliative, since these patients are diagnosed faster due to the disease progression, while patients who opt for curative treatments undergo more tests to make a more accurate diagnosis, which lengthens waiting times [21].

For the diagnosis-to-treatment time, nine studies reported improved survival when the waiting time was shorter, three studies found no association between waiting time and survival or mortality, and nine studies reported improved survival when the waiting time was longer; in these studies the results obtained were justified by indicating that patients in more advanced stages, or who are older or with worse health are referred and treated more quickly than those in earlier stages, whose diagnosis may require more tests that delay the time to treatment, and in whom, despite being treated more quickly, due to the disease severity, the poor prognosis is not altered. In addition, the studies clarified that, despite these results, the timely treatment of patients with early-stage SCLC should be emphasized to prevent a worsening in staging, which has a large impact on survival [18, 22].

## Discussion

We analyzed 38 articles on waiting times for the diagnosis and treatment of lung cancer published between 2010 and 2020 which related them to the prognosis. The studies selected were widely heterogeneous in terms of the design, the patient populations included, the structure of the health systems, the definition of the waiting time intervals evaluated, and the summary statistics used in the analysis of the results, which limits possible between-study comparisons.

In similar studies, Olsson 2019 [6] reported a range of medians for the diagnosis to treatment time of 12.5–52 days, and from primary care visit to specialist visit time of 1–12 days; Jacobsen et al. [1] found a median range of 6–45 days and 1–17 days, respectively, for the same time intervals. In our review, medians of 6–121 days were found for the diagnosis to treatment time and 4–19.5 days for the primary care visit to specialist visit time, suggesting that waiting times have not improved and efforts should be made to reach the recommended standard times of a median of 15 days between diagnosis and treatment and 7 days between the primary care visit and the specialist visit [7].

We found that 35% of the time intervals studied showed no relationship between mean or median waiting times and the disease prognosis. Paradoxically, in the rest of the times studied, 37.5% found a better prognosis with longer waiting times and 27.5% a better prognosis with shorter waiting times. Jacobsen et al. [1] and Olsson [6] also obtained disparate results in terms of the proportion of articles that related better patient prognosis with longer times, shorter times, or that the prognosis was not affected by the waiting times, although in these reviews the results were not related to the specific waiting times, but a general evaluation of the relationship was made.

Although the results show that a high proportion of studies associated prolonged waiting times with a better prognosis, all of them justify this association, arguing for the importance of early care and detection in more serious patients. This suggests that to achieve a good management and prognosis of lung cancer these waiting times must be reduced. Most articles which associated shorter waiting times with a worse prognosis justified this relationship by stating that patients in more advanced stages, or who were older or had comorbidities, are referred and treated more quickly than those who are in earlier stages; in these more advanced patients, despite being treated more quickly, the poor prognosis did not change, resulting in shorter survival times. The diagnosis of patients in early stages may require more testing or evaluation by hospital committees, which delays diagnostic and treatment times, but may improve the prognosis because treatment is more targeted and individualized. In addition, many patients will receive surgical treatment, and the time spent on the waiting list until surgery can help prolong these intervals.

Special attention should be paid to the psychological stress to which patients are subjected throughout the process from diagnosis to treatment. As shown by Labbe et al. and Kasymjanova et al. [28, 32], shorter waiting times have positive repercussions in terms of anxiety, mental health, quality of life and patient satisfaction, and lead to lower treatment costs.

The global situation, in which COVID-19 has impacted on cancer waiting times in general, and lung cancer in particular, should be considered. Gheorghe et al. [38], modeled the potentially avoidable deaths due to delays in cancer diagnosis in England in response to the pandemic and estimated the economic and quality of life lost. Nearly 3620 deaths due to breast, bowel, lung, and esophageal cancer could have been avoided in the next 5 years, representing a loss of 32,700 QALYs and €120. 83 million in productivity and, specifically in lung cancer, 10,900 QALYs and €4.45 million, compared with the 21,450 QALYs and €88.96 million lost due to deaths caused by COVID-19. Therefore, good coordination and early action in the management of lung cancer patients is essential to alleviate the delays and consequences derived from COVID-19.

One limitation of our study is the variation in the countries of the studies selected and the differences in health systems, which has a direct impact on waiting times and can cause confusion, as does the differing measures of waiting times, since each article defines these differently, which impacts on the comparability of the results and the complexity of the interpretation. However, we used PubMed and Embase to extract most available studies on the objective, thus providing an overview of the waiting times lung cancer patients are subject to and a detailed analysis of these times with the prognosis.

Generally, the waiting times usually include biases. The times are not accelerated if the patient is in the earlier disease stages, but they are in the advanced stages, due to the high mortality in this type of cancer, which results in contradictory results. As indicated by Adizie et al. [39], there are also more factors that skew waiting times, such as physician’s workloads and the organization of the treating center, which negatively affect the survival of lung cancer patients, the type of curative treatment administered and reductions in waiting times. Further prospective evidence is required to enable studies designed to provide more data on the relationship between waiting times and lung cancer prognosis.

In conclusion, patients value timely and effective care, and it is important to improve the diagnostic and therapeutic waiting times to which lung cancer patients are subjected, especially because these times influence the prognosis, with the aim of increasing the cure rate or, where appropriate, improving the quality of life and prolonging survival.

## Data Availability

The datasets generated and/or analyzed during the current study are available from the corresponding author upon reasonable request.

## Appendix A

See Tables 2 and 3.

**Table 2:** Characteristics of the studies included

Abbreviated reference and country	Study design, patients included	Study duration (follow-up time)	Type of cancer (stage)	Inclusion criteria	Interesting results
Sheinson 2020 USA	Retrospective, 442 patients	7 years (NA)	SCLC (IIIB–IV)	ALK + SCLC, ALK + determination < 90 days and ALK inhibitor initiation < 90-days	HR, diagnosis to treatment time
Bhandari 2020 USA	Prospective, 64,491 patients	4 years (death/last follow-up date)	SCLC (I–IV)	≥ 18 years SCLC. Excluded if not received chemotherapy, initiation of chemotherapy > 180 days or if information was missing	Survival, diagnosis to initiation of chemotherapy time
Rice 2020 USA	Retrospective, 355 patients	13 years (5 years)	NSCLC (III)	NSCLC, with curative intent with neoadjuvant chemo-radiotherapy ± surgery	OS and recurrence-free ratio at 5 years, diagnosis to treatment time
Odell 2019 USA	Retrospective, 141,723 patients	15 years (2 years)	SCLC (I–IV)	NSCLC, with curative intent registered with the NCDB	HR, chemotherapy to adjuvant surgery time and surgery to adjuvant chemotherapy
Crawford 2020 USA	Retrospective, 3866 patients	3 years (death/last follow-up date)	NSCLC (III–IV)	≥ 18 years NSCLC, with first-line platinum-based chemotherapy	OS and relationship with dose delays and/or dose reduction, time to chemotherapy administration
Bhandari 2019 USA	Retrospective, 2992 patients	3 years (7.5 months, 12 and 24 months)	SCLC (I–IV)	≥ 18 years SCLC	Survival, diagnosis to treatment time
Alanen 2019 Finland	Retrospective, 221 patients	1 year (1 year)	Lung cancer (I–IV)	Lung cancer	OS, symptoms to treatment time and setbacks: until medical visit, until diagnosis, until treatment
Cushman 2020 USA	Retrospective, 140,455 patients	11 years (NA)	SCLC (I–III)	> 18 years NSCLC with curative intent registered in the NCDB	OS and time of initiation of treatment
Kuroda 2018 Japan	Retrospective, 293 patients	5 years (5 years)	SCLC (IA)	NSCLC with surgery at a referral center CT diagnosis to surgery > 6 months	OS and DFS at 5 years after CT detection. Times since CT detection and surgery
Malalasekera 2018 Australia	Prospective, 128 patients	NA	Lung cancer (I–IV)	Adults NSCLC or SCLC	Diagnosis to treatment times, related survival
Ha 2018 USA	Retrospective, 177 patients	7 years (NA)	Lung cancer (I–IIIA)	Lung cancer patients eligible for curative intent therapy	Diagnosis to treatment times, related survival
Anggondowati 2020 USA	Retrospective, 691,464 patients	8 years (5 years)	SCLC (I–IV)	≥ 18 NSCLC who underwent surgery, chemotherapy or radiation	Diagnosis to treatment times; OS and 5-year survival
Hanaoka 2018 Japan	Retrospective, 283 patients	14 years (5 and 10 years)	NSCLC (I–IIIB)	NSCLC	diagnosis to treatment time, survival ratios
Labbé 2017 Canada	Retrospective, 1251 patients	1.5 years (death/last follow-up date)	Lung cancer (I–IV)	Patients referred to the diagnostic evaluation program of the University Institute of Cardiology and Pulmonology of Quebec September 22, 2013–March 7, 2015	Waiting times for research and treatment; PFS, RFS after primary surgical resection, and OS
Vinod 2017 Australia	Retrospective, 1926 patients	6 years (NA)	SCLC (I–IV)	SCLC in the South Western Sydney Cancer Registry	Diagnosis to treatment time, mortality
Jacobsen 2017 USA	Systematic review (NA)	11 years (NA)	All (I–IV)	PubMed studies 2007–2016, reporting waiting times	Waiting times and survival, resource utilization, costs and inequalities
Kasymjanova 2017 Canada	Retrospective, 721 patients	5 years (1 year)	Lung cancer (I–IV)	Lung cancer	Multiple timepoints; survival
Yang 2017 USA	Retrospective, 4984 patients	5 years (death/last follow-up date)	NSCLC with squamous cell carcinoma (I)	Squamous cell carcinoma in the NCDB treated with lobectomy without chemotherapy or radiation	Diagnosis to treatment time, survival ratios
Salazar 2017 USA	Retrospective, 31,474 patients	9 years (5 years)	SCLC (I–III)	NSCLC underwent lobectomy or pneumonectomy treated with multi-agent chemotherapy, and who did not receive adjuvant chemotherapy	Resection to chemotherapy time; OS
Jing 2016 China	Prospective, 362 patients	7 years (death/last follow-up date)	SCLC (II–IIIA)	NSMP undergoing lobectomy and adjuvant chemotherapy	OS and DFS, time from surgery to treatment
Yennurajalingam 2017 USA	Retrospective, 410 patients	1 year (1 year)	SCLC (IIIB–IV)	NSDC in palliative care	OS, diagnosis to treatment time
Coughlin 2015 Canada	Retrospective, 222 patients	1 year (1 year)	NSCLC (I–II)	NSCLC stages I-II, operated between 2010–2011	OS, decision on surgery to surgery time
Van 2015 Australia	Retrospective, 713 patients	2 years (death/last follow-up date)	Lung cancer (I–IV)	Lung cancer	Times between first abnormal diagnostic image, specialist consultation, biopsy, remission, oncology consultation and initiation of treatment, OS
Gómez 2015 USA	Retrospective, 28,732 patients	3 years (1 year)	SCLC (I–IV)	≥ 66 years NSCLC	Prevalence of delay in treatment, patient components, disease and medical supply that contributed to the delay in treatment, and effect of delay on survival, benchmarks on the timeliness of staging studies which could significantly reduce delays
Redaniel 2015 UK	Retrospective, 5737 patients	11 years (5 years)	Lung cancer (NA)	≥ 15 years breast, colorectal, lung, and prostate cancer	Time to diagnosis; related survival
Samson 2015 USA	Retrospective, 55,653 patients	12 years (NA)	SCLC (I)	NSCLC undergoing pulmonary resection	Delays in time to surgery; survival
Zicovic 2014 Montenegro	Retrospective, 206 patients	2 years (1 year)	Lung cancer (NA)	Lung carcinoma	Symptoms to primary care visit, primary care visit to diagnosis, visit to specialist to diagnosis; related survival
Forrest 2015 UK	Retrospective, 23,497 patients	3 years (2 years)	Lung cancer (I–IV)	Lung cancer	Survival, diagnosis to treatment times
Kanarek 2014 USA	Retrospective, 174 patients	6 years (1 year)	NSCLC (I–II)	Patients undergoing surgery for early-stage NSCLC at SKCCC	Diagnosis to first surgery time; related survival
González-Barcala 2013 Spain	Retrospective, 307 patients	3 years (death)	Lung cancer (I–IV)	Lung cancer	Delays in diagnosis and treatment; related forecast
Radzikowska 2013 Poland	Retrospective, 3479 patients	3 years (5 years)	SCLC (I–IV)	SCLC	Survival, delays caused to patients and inactivity of doctors, dates of first contact with a specialist doctor and first bronchoscopy
Concannon 2020 USA	Retrospective, 133 patients	6 years (NA)	SCLC (I–IV)	NSCLC	OS, finding of suspected nodule by image until confirmation diagnosis (biopsy) and between diagnosis and first treatment times
Booth 2013 Canada	Substudy retrospective, 3354 patients	2 years (4 years)	SCLC (I–IV)	NSCLC time to surgical resection ≤ 24 weeks of diagnosis	Surgery to treatment and diagnosis to treatment times, related survival
Radzikowska 2012 Poland	Retrospective, 10,386 patients	3 years (death/last follow-up date)	SCLC (I–IV)	NSCLC	Dates of the first specialist visit and first bronchoscopy and associated survival
Diaconescu 2011 Canada	Retrospective, 665 patients	2 years (3 years)	SCLC (NA)	NSCLC	Delay in treatment, associated survival
Skaug 2011 Norway	Retrospective, 271 patients	6 years (1, 2, 3, 4, 5, 10 years)	Lung cancer (I–IV)	Lung cancer residents of the central area	OS, first symptom to diagnosis time
González-Barcala 2010 Spain	Retrospective, 481 patients	3 years (NA)	Lung cancer (I–IV)	Lung cancer	Delay in the patient's consultation and the diagnostic and therapeutic process; associated survival
Wah 2020 Australia	Retrospective with spatial modeling, 3300 patients	5 years (2 years)	SCLC (I–IV)	> 18 years NSCLC in the Victoria Lung Cancer Registry	Risk of any-cause death related to the time from diagnosis to treatment

CT, computerized axial tomography; DFS, disease-free survival; HR, hazard ratio; NA, not available; NCDB, National Cancer Database; NSCLC, non-small cell lung cancer; OS, overall survival; PFS, progression-free survival; RFS, relapse-free survival; SCLC, small cell lung cancer

**Table 3:** Waiting times and association with survival

	Abbreviated reference (author. year), and *n* of patients	Type of cancer	Stage	Time interval	Definition of delay according to article	Group	% patients	Mean (days)	SD	Median (days)	IQR	Minimum–maximum	Prognosis
Symptoms to treatment	Alanen 2019 221 patients	Lung cancer	I–IV	First symptom to treatment	Time greater than the median	–	–	–	–	130.5	–	20–488	No association. In stage I survival improves if the time interval is shorter
	González-Barcala 2010 481 patients	Lung cancer	I–IV	Symptoms to treatment	–	–	–	123.6	141.6	87.5	53–139	–	Improves survival with longer times
	Radzikowska 2013 3479 patients	SCLC	I–IV	Symptoms to treatment	–	–	–	113.3	118.5	78	45–135	–	No association
	Diaconescu 2011 665 patients	SCLC	Nd	First abnormal image to treatment	–	–	–		–	62	30–108	–	Improves survival with longer times for advanced disease, in the regional-local group survival is not related to waiting times
	Kuroda 2018 293 patients	SCLC	AI	Suspected CP to surgery	> 24 months after CT detection					882	–	–	Improves survival with longer times
Symptoms to first specialist visit	González-Barcala 2013 307 patients	Lung cancer	I–IV	Symptoms to first specialist visit	–	–	–	53.6	–	53	–	–	No association
	González-Barcala 2010 481 patients	Lung cancer	I–IV	Symptoms to first specialist visit	–	–	–	82.1	133.8	48.5	22–92.7	–	No association
Symptoms at first medical visit	Radzikowska 2012 10,386 patients	SCLC	I–IV	First symptom to first medical visit		–	–	56.7	–	–	–	–	Improves survival with shorter times
	Alanen 2019 221 patients	Lung cancer	I–IV	First symptom to first medical visit	Time greater than the median	–	–	–	–	58	–	6–428	No association in general. Survival in stage I improves if time interval is shorter
	Radzikowska 2013 3479 patients	SCLC	I–IV	Symptoms to first medical visit	–	–	–	47.4	80.3	30	9–59	–	No association
	Zicovic 2014 206 patients	Lung cancer	NA	Symptoms to primary care visit	–	–	–	43.4	–	28	–	1.61–231	Non-significantly survival improves with longer times
S ymptoms to diagnosis	Alanen 2019 221 patients		I–IV	First medical visit to diagnosis	Time greater than the median	–	–	–	–	33	–	− 8 to 215	Improves survival with longer times
	Skaug 2011 271 patients	Lung cancer	I–IV	First symptom to diagnosis	–	–	–	–	–	66	32–113	–	No association
	Concannon 2020 133 patients	SCLC	I–IV	Abnormal biopsy/diagnostic imaging	50 days	Stages I–II homeless		248	–	–	–	–	No association
		SCLC	I–IV	Abnormal biopsy/diagnostic imaging	50 days	Stages I–II not homeless		116	–	–	–	–	No association
		SCLC	I–IV	Abnormal biopsy/diagnostic imaging	50 days	Stages III–IV homeless		34.7	–	–	–	–	No association
		SCLC	I–IV	Abnormal biopsy/diagnostic imaging	50 days	Stages III–IV not homeless		46	–	–	–	–	No association
First medical visit to diagnosis	Redaniel 2015 5737 patients	Lung cancer	NA (C34 ICD 10 (Malignant neoplasm of bronchus and lung.))	Primary care visit to diagnosis	–	–	–	–	–	88	34–210	–	Improves survival with longer times,
	Zicovic 2014 206 patients	Lung cancer	Nd	Primary care visit to diagnosis	–	–	–	29.54	–	–	–	7–161	No significantly improved survival with shorter times
First specialist visit to diagnosis		Lung cancer	Nd	Specialist visit to diagnosis	–	–	–	16.59	–	–	–	–	No association
	González-Barcala 2013 307 patients	Lung cancer	I–IV	First specialist visit to diagnosis	–	–	–	31.5	–	18	–	–	No association
	Ibbé. 2017 1251 patients	Lung cancer	I–IV	Referral to specialist to diagnosis	–	–	–	–	–	21	13–37	–	No association
Diagnosis to treatment	Ha. 2018 177 patients	Lung cancer	I–IIIA	Diagnosis to treatment	–	–	–	35	–	–	–	–	No association
	González-Barcala 2013 307 patients	Lung cancer	I–IV	First specialist visit to treatment	–	–	–	55.2	–	35	–	–	Improves survival with longer times
	González-Barcala 2010 481 patients	Lung cancer	I–IV	First specialist visit to treatment	–	–	–	41.1	52.9	27	17–42	–	Improves survival with longer times
	Labbé. 2017 1521 patients	Lung cancer	I–IV	Referral to specialist to first treatment		–	–	–	–	56	34–81	–	No association
		Lung cancer	I–IV	Referral to specialist to surgery	–	–	–	–	–	70	51–91	–	No association
	Forrest 2015 23,497 patients	Lung cancer	I–IV	Referral to specialist to treatment	> 14 days	≤ 14 days	0.307	–	–	–	–	–	Improves survival with longer times, in stages III and IV
		Lung cancer	I–IV	Referral to specialist to treatment	> 14 days	> 14 days	0.693	–	–	–	–	–	Improves survival with longer times, in stages III and IV
	Alanen 2019 221 patients	Lung cancer	I–IV	Diagnosis to treatment	Time greater than the median	–	–	–	–	27	–	− 21 to 148	Improves survival with longer times
	Kasymjanova 2017 751 patients	Lung cancer	I–IV	Diagnosis to treatment	–	treatment	–	–	–	27	5–45	–	Improves survival with shorter times
		Lung cancer	I–IV	Diagnosis to treatment	–	chemotherapy	–	–	–	32	20–46	–	Improves survival with shorter times
		Lung cancer	I–IV	Diagnosis to treatment	–	radiotherapy	–	–	–	35	19–55	–	Improves survival with shorter times
	Forrest 2015 23,497 patients	Lung cancer	I–IV	Diagnosis to treatment	> 31 days	≤ 31 days	0.605	–	–	–	–	–	Improves survival with longer times, in stages III and IV
		Lung cancer	I–IV	Diagnosis to treatment	> 31 days	> 31 days	0.395	–	–	–	–	–	Improves survival with longer times, in stages III and IV
	González-Barcala 2013 307 patients	Lung cancer	I–IV	Diagnosis to treatment	–	–	–	23.5	–	14	–	–	Improves survival with longer times
	Samson 2015 55,653 patients	SCLC	I	Diagnosis to surgery	< 8 weeks	–	–	–	–	29	20–41	–	Improves survival with shorter times
		SCLC	I	Diagnosis to surgery	8 weeks or more	–	–	–	–	77	64–102	–	Improves survival with shorter times
	Kanarek 2014 174 patients	SCLC	I–II	Diagnosis to Surgery	–	–	–	67.2		–	–	–	Improves survival with shorter times
	Sheinson 2020 442 patients	SCLC	IIIB–IV	Diagnosis to treatment	21 days	–	–	–	–	21	–	–	Shorter time less mortality
	Cushman 2020 14,055 patients	SCLC	I–III	Diagnosis to treatment	45 days	–	–	–	–	28	–	1–365	Improves survival with shorter times
	Concannon 2020 133 patients	SCLC	I–IV	Biopsy to treatment	–	Stages I–II homeless	–	20	–	–	–	–	No association
		SCLC	I–IV	Biopsy to treatment	–	Stages I–II not homeless	–	50	–	–	–	–	No association
		SCLC	I–IV	Biopsy to treatment	–	Stages III–IV homeless	–	49.9	–	–	–	–	No association
		SCLC	I–IV	Biopsy to treatment	–	Stages III–IV not homeless	–	58.1	–	–	–	–	No association
	Wah 2020 3300 patients	SCLC	I–IV	Diagnosis to treatment	> 14 days	> 14 days	0.5	–	–		–	–	Shorter time less mortality
	Anggondowati 2020 691,464 patients	SCLC	I–IV	Diagnosis to treatment	–	Metastases	–	–	–	18	11–36	–	No association
		SCLC	I–IV	Diagnosis to treatment	–	Early stages	–	–	–	28	2–51	–	Shorter time less mortality
		SCLC	I–IV	Diagnosis to treatment	–	Local disease	–	–	–	27	13–46	–	No association
	Vinod 2017 1926 patients	SCLC	I–IV	Diagnosis to treatment	–	–	–	–	–	32	15–18	–	Improves survival with longer times, in stages III and IV
	Gomez 2015 28,732 patients	SCLC	I–IV	Diagnosis to treatment	> 35 days	Local disease	–	–	–	–	–	–	Improves survival with shorter times
		SCLC	I–IV	Diagnosis to treatment	> 35 days	Regional disease	–	–	–	–	–	–	No association
	Radzikowska 2012 10,386 patients	SCLC	I–IV	Diagnosis to treatment	–	–	–	32		0		–	Improves survival with longer times
	Yang 2017 4984 patients	NSCLC with squamous cell carcinoma	I	Diagnosis to treatment	–	–	–	–	–	38	24–60	–	Improves survival with shorter times
	Bhandari 2020 64,491 patients	SCLC	I–IV	Diagnosis to treatment	–	–	–	–	–	18	9–31	–	Improves survival with longer times,
	Bhandari 2019 2992 patients	SCLC	I–IV	Diagnosis to treatment	–	–	–	18	–		–	–	Improves survival with longer times,
	Radzikowska 2013 3479 patients	SCLC	I–IV	Diagnosis to treatment	–	–	–	28.9	64,4	6	6–21	–	Improves survival with longer times
Decision on surgery to surgery	Coughlin 2015 222 patients	SCLC	I–II	Decision on surgery to surgery	–	< 1 month	0.248	–	–	–	–	–	No association
		SCLC	I–II	Decision on surgery to surgery	–	1 < 2 months	0.441	–	–	–	–	–	No association
		SCLC	I–II	Decision on surgery to surgery	–	2 < 3 months	0.19	–	–	–	–	–	Improves survival with shorter times in stage II; No association for stage I
		SCLC	I–II	Decision on surgery to surgery	–	3 < 4 months	0.117	–	–	–	–	–	No association
Diagnosis to first specialist contact	Kanarek 2014 174 patients	SCLC	I–II	Diagnosis to first contact with the center		–	–	61.2	–	–	–		Improves survival with shorter times
Surgery to treatment/chemotherapy	Odell 2019 141,723 patients	SCLC	I–IV	Surgery to chemotherapy	> 180 days	–	–	0.665	–	NA	–	–	Improves survival with shorter times
		SCLC	I–IV	Chemotherapy to surgery	> 120 days	–	–	0.037	–	–	–	–	Improves survival with shorter times
	Salazar 2017 31,474 patients	SCLC	I–III	Surgery to treatment	–	–	–	–	–	48	37–62	–	No association
	Jing 2016 362 patients	SCLC	II–IIIA	Surgery to treatment	8 weeks	–	–	–	–	48	–	26–102	Improves survival with shorter times
	Booth 2013 3354 patients	SCLC	I–IV	Surgery to treatment	–	–	–	–	–	56	–	7–112	No association

NSCLC, non-small cell lung cancer; SCLC, small cell lung cancer; NA, not available; CT, computerized axial tomography
